# History of a Case Illustrative of the Good Effects of Blood-Letting in Puerperal Fever

**Published:** 1820-11

**Authors:** Thomas Aspray

**Affiliations:** Surgeon.


FOR THE LONDON MEDICAL AND PHYSICAL JOURNAL.

### *History of a Case illustrative of the good Effects of Blood-letting in Puerperal Fever.* By Mr. Thomas Aspray, Surgeon.

MRS. R—, aged 23, of spare habit, although of general good health, was delivered, on the 30th of July, after the ordinary term of utero-gestation, of a fine child, without any deviation from the usual circumstances of natural labour. The parturient process was of moderate duration; the secundines succeeded the child in about half an hour, and the patient appeared comfortable. However, about twelve o'clock at midnight of the 31st July, a marked cold rigor occurred, increasing in intensity until it became absolute quaking of the whole body. This remained half an hour, and was quickly followed by pain of the abdomen and head, accompanied with death-like pallidity diffused over the countenance, and great prostration of strength. At noon, the succeeding day, (August 1st,) the rigor recurred, though less in degree. This excited alarm, and I was consulted. In addition to the enumerated symptoms, I found quick pulse of 120, and considerable soreness on pressure of the abdominal surface; from which I deemed it advisable to extract blood, which was done to the quantity of twelve ounces. This mitigated the vehemence of pain, &c. ; but in the evening it increased, and a second bleeding was resorted to of the same amount, which also was productive of relief.

On the 2d of August, febrifuge medicines, as liq. ammon. acet. and p. Jacobi, quartis horis, were used, in order to determine to the surface and excite perspiration; which was soon effected, with the aid of regulation of the bed-clothes.

---

As the pain continued, although somewhat abated, twenty leeches were applied to the abdomen, which seemed to produce but little benefit. This induced me again to bleed; accordingly, ten ounces more blood were removed, with considerable alleviation of the patient's suffering.

*August* 3.—Much pain is present: ten ounces more of blood were abstracted, and febrifuge and purgative medicines were administered; which last procured copious evacuations.

*Aug.* 4.—The disease seems so far reduced, that only slight sensations of an unpleasant kind exist. Diaphoretic plan continued.

*Aug.* 5, 6, 7, 8, and 9.—Patient is recovering.

*Aug.* 10.—Febrifuges and slight tonics prescribed.

*Aug.* 12.—Ext. cinchonæ used, and carbonate of ammonia.

*Aug.* 13 and 14.—Still mending, although slight occasional grinding pains occur.

*Aug.* 15.—The whole train of symptoms is renewed, from the abuse of brandy by an injudicious nurse. This inference is obvious, as the relapse came on in two or three hours after drinking two table-spoonfuls of spirit; and indeed pain was felt immediately after that unfortunate, though commonly-resorted-to, potation of nurses. The case was now one of fearful issue; for, from antecedent depletion having much reduced the patient, the adoption of a similar practice must have been at variance with the indications of the common institutes for the practice of physic: therefore, agreeably with the depressed state of the system, leeches were applied to the abdomen; and, as there had been no free passage through the bowels, a few grains of submuriate of mercury were given, followed by a saline aperient draught. This combination freely opened the intestinal passage, after the exhibition of a laxative clyster. The sudorific plan was persisted in, and a large blister applied over the whole belly: this induced most distressing strangury, which easily yielded to hot applications and diluents of a mucilaginous kind. Since this, the morbid signs have diminished, and the patient is rapidly advancing to perfect convalescence.

It is very evident that this case is what has been termed *sporadic puerperal fever*, and hence a milder form of the complaint: yet there were symptoms of typhoid condition in this case, which, with the foregoing account, would present an analogy of such closeness, as to suggest that the success of a plan in the one case would indicate its propriety in the other; although, of course, it must be early resorted to: and it would appear that this case tends to show the futility of attempting to reduce medical reasoning to mathematical accuracy, as is the case in some treatises, by laying down as an axiom, "that what will not prevent will not cure," from the observation that profuse hemorrhages from the uterus during labour have not prevented subsequent puerperal fever; therefore bleeding, it is said, cannot avail in cure. It was the opinion of a late eminent surgeon in London, that bleeding would not prevent inflammation.

*Olney; Sept.* 1820.

